# The application of sparse estimation of covariance matrix to quadratic discriminant analysis

**DOI:** 10.1186/s12859-014-0443-6

**Published:** 2015-02-18

**Authors:** Jiehuan Sun, Hongyu Zhao

**Affiliations:** Department of Biostatitics, Yale School of Publich Health, 60 College Street, New Haven, 06511 CT USA

**Keywords:** Quadratic discriminant analysis, Sparse estimation of covariance matrix, Classification, Genomics

## Abstract

**Background:**

Although Linear Discriminant Analysis (LDA) is commonly used for classification, it may not be directly applied in genomics studies due to the large *p*, small *n* problem in these studies. Different versions of sparse LDA have been proposed to address this significant challenge. One implicit assumption of various LDA-based methods is that the covariance matrices are the same across different classes. However, rewiring of genetic networks (therefore different covariance matrices) across different diseases has been observed in many genomics studies, which suggests that LDA and its variations may be suboptimal for disease classifications. However, it is not clear whether considering differing genetic networks across diseases can improve classification in genomics studies.

**Results:**

We propose a sparse version of Quadratic Discriminant Analysis (SQDA) to explicitly consider the differences of the genetic networks across diseases. Both simulation and real data analysis are performed to compare the performance of SQDA with six commonly used classification methods.

**Conclusions:**

SQDA provides more accurate classification results than other methods for both simulated and real data. Our method should prove useful for classification in genomics studies and other research settings, where covariances differ among classes.

## Background

Although Linear Discriminant Analysis (LDA) is commonly used for classification, it cannot be directly applied in the large p, small n setting, where the number of samples is far less than the number of features [1]. This is due to the singularity of the sample covariance matrix.

One straightforward solution for the matrix singularity problem is to use the generalized inverse, e.g. the Moore-Penrose pseudo-inverse, as mentioned in [2-4] in deriving the LDA rule. Alternatively, several authors have proposed modified estimators of the covariance matrix to address the singularity problem, among which the most common approach is to shrink the sample covariance matrix towards a well-behaved matrix. For example, penalized discriminant analysis was proposed in [5], where the sample covariance matrix is shrunken towards the identity matrix. It has also been proposed to shrink the sample covariance matrix towards a common diagonal covariance matrix or simply use the diagonal covariance matrix [6,7]. The relative performance of using sample covariance matrix, common covariance matrix, diagonal sample covariance matrix, and diagonal common covariance matrix as an estimator for the covariance matrix in discriminant analysis was studied in [6]. Another version of the penalized discriminant analysis was proposed in [8], where the shrunken estimator of covariance matrix proposed in [5] was combined with the application of “nearest shrunken centroids” proposed in [9] to estimate the sample means. A modified linear discriminant analysis was later proposed in [10], where a well-conditioned estimator for high-dimensional covariance matrix proposed in [11,12] was used. Other authors have addressed the singularity problem through the eigenvalue decomposition by discarding small eigenvalues [3,13]. Most recently, progress on sparse estimation of covariance matrix and precision matrix offers another approach for LDA in the large p, small n setting where sparsity assumption is imposed on the covariance matrix or precision matrix. This class of methods is called Sparse LDA, where there are different ways of imposing sparsity on the covariance matrix and/or mean difference through different optimization methods [14-16].

Quadratic discriminant analysis (QDA) is closely related to LDA, where each class is assumed to have its own covariance matrix. This is in contrast to LDA where the covariance matrices for different classes are assumed to be the same. For many practical problems, the assumption of common covariance matrix across different classes is inappropriate. For example, in the case of using gene expression data to distinguish tumor samples from normal controls, or to distinguish tissue types, many studies have shown that the correlation patterns among genes do differ between cancer and normal samples and among tissues. In fact, such rewiring of genetic networks in patients and across tissues are common and sometimes offer valuable information for classifications [17-19]. These observations suggest that the covariance or precision matrices of different classes are different and QDA may be more appropriate than LDA for classifications in these settings.

Although many efforts have been made to improve LDA in high-dimensional settings, as far as we are aware, relatively little has been done to improve QDA for the large p, small n problem. This is partly due to the fact that it is already challenging to estimate the covariance or precision matrix in the high-dimensional setting when all the classes are assumed to have the same form for LDA, it will be more difficult to estimate covariances in QDA, since more parameters need to be estimated where each class is allowed to have a different covariance matrix. In this paper, we propose a novel QDA based classification method that estimates separate sparse covariance matrices for different classes, called SQDA, and compare its performance with existing methods, including diagonal linear discriminant analysis (DLDA), diagonal quadratic discriminant analysis (DQDA), regularized discriminant analysis with shrunken centroids (SCRDA), nearest neighbor (NN) [20], support vector machine (SVM) [21], and random forests (RF) [22], where NN, SVM and RF are commonly used in genomics studies. The authors of [23] proposed a related method on sparse QDA, in which joint estimation of precision matrices for different classes is applied to each block determined by hierarchical clustering based on the correlation matrices, where block diagonal structure is imposed on the covariance matrices. However, their method is computationally more involved in high-dimensional settings and hence we didn’t include their method in the comparisons.

## Results

### Simulations

For our proposed method, the covariance matrices for different classes are assumed to be block-diagonal with each block having the same size and variable selection by blocks is performed. For block-diagonal matrices, the block size has to be determined. For variable selection by blocks, the blocks with cross validation errors exceeding the smallest error among all blocks by predefined error margin will be excluded.

In this section, we study the effects of block size, error margin, and sample size on our method. We also compare the performance of our method with other classification methods under different simulation settings. The detailed simulations can be found in the Materials and Methods section.

#### Simulation settings

In the first simulation setting, Independent Structure Same Covariance (ISSC), the covariance matrices are the same and diagonal for all classes. We let the number of genes be 10,000 and generate 100 training samples consisting of 50 “tumor” and 50 “normal” samples. The gene expression values in the tumor samples are drawn from *N*(*μ*_1_,*Σ*_1_) and those in the normal samples are drawn from *N*(*μ*_2_,*Σ*_2_), where *Σ*_1_=*Σ*_2_=*I* are the identity matrix. We assume that the first 400 elements in *μ*_1_ are 0.5 and the rest are 0 while all elements in *μ*_2_ are 0. The testing dataset has 500 tumor samples and 500 normal samples.

In the second simulation setting, Independent Structure Different Covariance (ISDC), the set-up is the same as above except that *Σ*_1_ is changed from the identity matrix with the first 400 diagonal elements replaced by 1.5.

The third simulation setting, Dependent Structure Same Covariance (DSSC), puts some dependences on the genes. More specifically, *Σ*_1_ and *Σ*_2_ have the same blockwise autoregression correlation structure *Σ*

(1) $$ {\fontsize{9.5pt}{9.6pt}\selectfont{\begin{aligned} \Sigma= \left(\begin{array}{cccccc} \Sigma_{\rho} & 0 & 0 &\cdots&\cdots&\cdots \\ 0 & \Sigma_{-\rho} & 0 &0&\cdots&\vdots \\ 0 & 0 &\Sigma_{\rho} &0 &\cdots&\vdots\\ \vdots &0& 0 &\Sigma_{-\rho} &0 &\vdots\\ \vdots &\vdots& \vdots &0 &\ddots &\vdots\\ \cdots &\cdots& \cdots &\cdots &\cdots &\cdots\\ \end{array} \right)_{10,000\times 10,000} \end{aligned}}}  $$

and

(2) $$ {\fontsize{9.7pt}{9.6pt}\selectfont{\begin{aligned} \Sigma_{\rho}= \left(\begin{array}{ccccc} 1& \rho& \cdots&\rho^{198}&\rho^{199} \\ \rho& 1 & \ddots &\cdots&\rho^{198} \\ \vdots &\ddots& \ddots &\ddots &\vdots\\ \rho^{198} &\cdots& \ddots &\ddots &\rho \\ \rho^{199}&\rho^{198}& \cdots &\rho&1\\ \end{array} \right)_{200\times 200} \end{aligned}}}  $$

where *ρ*=0.95 and *Σ*_−*ρ*_ has the same structure as *Σ*_*ρ*_, but with -0.95 for *ρ*.

In the fourth simulation setting, Dependent Structure Different Covariance (DSDC), *Σ*_1_ and *Σ*_2_ are the blockwise autoregression correlation structure as in Equation (1) except that the order of *Σ*_*ρ*_ and *Σ*_−*ρ*_ is reversed in *Σ*_2_ for the first two blocks.

For each simulation setting, we generate 10 datasets and the misclassification rates are reported as the average of the errors on the 10 testing datasets. The standard deviations of the misclassification rates are also reported together with the number of features selected for classification.

#### Effects of block size and error margin

We study the effects of two important parameters, block size and error margin, on the performance of SQDA.

**Figure 1:**
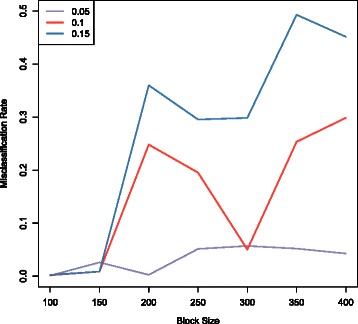
**The effects of block size and error margin on SQDA.** The effects of two important parameters, block size and error margin, on SQDA are shown in this figure based on the simulated data.

For the fourth simulation setting DSDC, we compare the performance of SQDA under different block sizes and error margins. The block sizes are varied from 100, 150, 200, 250, 300, 350, to 400, and the error margins are varied from 0.05, 0.10, to 0.15. The average misclassification rates under different combinations of block sizes and error margins are shown in Figure 1. We can see that when the block size is 100, the misclassification rates are consistently low for different error margins. Similarly, the misclassification rates are low for different block sizes when the error margin is 0.05. The optimal block size depends on the true underlying block structure in the data, which is 200 in simulations. It can be seen that the performance of our method is satisfactory when the block size is 100. Also, the optimal error margin depends on the sample size. When the sample size is small, where the cross validation error itself has large variation, a larger error margin is preferred to include more predictive features. In contrast, when the sample size is large, a smaller error margin might be better to exclude pure noises. For simplicity, we use block size 100 and error margin 0.05 for all later analyses.

#### Effect of sample size

The performance of any classification depends on sample size, which may be especially so for our method, since the number of parameters to be estimated is large and low sample size may lead to unstable results. To study the effect of sample size on our method and other methods, all the parameters are kept the same except that the sample size is varied among 20, 25, 30, 35, 40, 45 and 50 under the fourth simulation setting.

**Figure 2:**
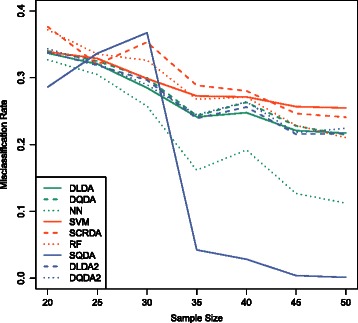
**The effect of sample size on the seven classification methods.** The effect of sample size on SQDA and six other classificaiton methods is shown in this figure based on the simulated data.

The average misclassification rates are shown in Figure 2 for all methods. We consider variable selection by blocks for DLDA2 and DQDA2 the same as in our method except that the sparse estimation of covariance matrix is replaced with diagonalized estimator for covariance matrix. By comparing the performance of SQDA to DLDA2 and DQDA2, we can see the benefit of sparse estimation of different covariance matrices for different classes in addition to that from the variable selection by blocks procedure. It is clear that the performance of all methods is equally poor when the sample size is small whereas the improvement is largest for our method when the sample size increases.

#### Performance of different methods on simulated data

In this part, we compare the performance of different methods on the simulated data, where we consider sample size of 50, block size of 100, and error margin of 0.05.

It is clear from Table 1 that our method has the best or close to the best performance among all the methods considered across different simulation scenarios.

**Table 1:** **Comparisons of seven classification methods on simulated data**

**Methods**	**ISSC**	**ISDC**	**DSSC**	**DSDC**
DLDA	0.048 (0.015, 50)	0.083 (0.015, 100)	0.228 (0.02, 1025)	0.217 (0.04, 1175)
DQDA	0.049 (0.021, 50)	0.013 (0.007, 50)	0.243 (0.025, 1400)	0.214 (0.032, 825)
NN	0.056 (0.02, 100)	0.424 (0.021, 50)	0.27 (0.034, 575)	0.112 (0.061, 475)
SVM	0.054 (0.029, 50)	0.095 (0.024, 100)	0.127 (0.047, 500)	0.255 (0.05, 1050)
SCRDA	0.019 (0.036, 651)	0.024 (0.012, 2089)	0.217 (0.041, 587)	0.241 (0.069, 317)
RF	0.109 (0.012, NA)	0.038 (0.009, NA)	0.262 (0.018, NA)	0.21 (0.041, NA)
SQDA	0.005 (0.002, 300)	0.001 (0.001, 300)	0.108 (0.042, 200)	0.001 (0.002, 400)
DLDA2	0.002 (0.001, 600)	0.04 (0.005, 500)	0.224 (0.03, 700)	0.217 (0.055, 600)
DQDA2	0.003 (0.001, 500)	0 (0.001, 600)	0.231 (0.033, 600)	0.224 (0.058, 400)

The reported numbers in each table entry in the form of *a (b,c)* mean: *a* is the average prediction error, *b* is the standard deviation, and *c* is the median number of predictors selected.

When the true covariance matrix is diagonal and the same for different classes (ISSC), DLDA2 performs the best and is much better than DLDA.

When the true covariance matrix is diagonal and different for different classes (ISDC), DQDA2 performs the best and is much better than DQDA.

Also, comparing the number of predictors selected by DLDA and DLDA2 or DQDA and DQDA2 suggests variable selection by the blocks method is favorable over variable selection by cross-validation. The number of predictors selected by cross-validation is either too small (50 or 100 for simple underlying covariance structure) or too large (1000+ for complex underlying covariance structure) while the number of predictors selected by variable selection by blocks is closer to the true number of predictors, which is 400 for all scenarios.

When the covariance structure is complex (DSSC and DSDC), the performance of SQDA is much better than all the other methods. The relative performance of SQDA with that of DLDA2 and DQDA2 suggests that the sparse estimation of different covariance matrix for different classes can indeed improve sample classification.

### Real data

In this section, we compare the performance of different classification methods on the TCGA dataset, which we downloaded from the UCSC cancer browser (https://genome-cancer.ucsc.edu), with the level 3 RNAseq data for all cancers log2-transformed and mean normalized across all cancer cohorts. We consider all cancer cohorts with at least 40 normal tissue samples, including Liver Cancer (LC, 191 tumor and 50 normal), Colon Cancer (CC, 262 tumor and 41 normal), Head and Neck Cancer (HNC, 497 tumor and 43 normal), Breast Cancer (BC, 1040 tumor and 112 normal), Lung Adenocarcinoma (LUAD, 488 tumor and 58 normal), Prostate Cancer (PC, 333 tumor and 50 normal), Kidney Clear Cell Carcinoma(KC, 518 tumor and 72 normal), Lung Squamous Cell Carcinoma (LUSC, 490 tumor and 50 normal) and Thyroid Cancer (TC, 498 tumor and 59 normal).

For all datasets, 20 tumor samples and 20 normal samples are randomly selected to be the training data and the remaining samples are treated as the testing data. Tuning parameters of all methods are selected using the training data and then the misclassification rates are estimated using the testing data. The whole process is repeated 10 times and the average misclassification rate and standard deviation for each method are reported together with the median number of features (i.e. genes) selected.

For real data analysis, we choose the block size to be 100 and error margin to be 0.05 for all datasets, as suggested by simulations. The results are shown in Table 2. We can see that SQDA performs best or is comparable to the best for all datasets except the PC datasets. Because the sample size for each class is 20, we increase the error margin to include more true signals. In fact, the performance of SQDA can improve when the error margin is 0.1 (results not shown).

**Table 2:** **Comparisons of seven classification methods on TCGA data**

**Methods**	**LUSC**	**LUAD**	**TC**
DLDA	0.013 (0.007, 50)	0.035 (0.027, 50)	0.1 (0.042, 50)
DQDA	0.008 (0.006,50)	0.018 (0.019, 50)	0.08 (0.047, 50)
NN	0.014 (0.01, 50)	0.027 (0.016, 50)	0.085 (0.053, 50)
SVM	0.01 (0.007, 50)	0.024 (0.017, 50)	0.088 (0.041, 50)
SCRDA	0.039 (0.031, 128)	0.044 (0.026, 95)	0.122 (0.068, 502)
RF	0.007 (0.002, NA)	0.018 (0.009, NA)	0.08 (0.039, NA)
SQDA	0.003 (0.003, 1900)	0.011 (0.009, 1900)	0.036 (0.021, 2900)
DLDA2	0.017 (0.005, 12100)	0.031 (0.013, 8900)	0.114 (0.038, 2300)
DQDA2	0.008 (0.004,10000)	0.023 (0.008, 8300)	0.107 (0.05, 5800)
**Methods**	**PC**	**HNC**	**LC**
DLDA	0.125 (0.024, 50)	0.034 (0.012, 50)	0.055 (0.017, 50)
DQDA	0.11 (0.022, 50)	0.03 (0.016, 50)	0.045 (0.021, 50)
NN	0.094 (0.029, 50)	0.032 (0.013, 50)	0.051(0.015, 50)
SVM	0.116 (0.031, 150)	0.037 (0.023, 50)	0.04 (0.014, 50)
SCRDA	0.094 (0.037, 1989)	0.039 (0.021, 2200)	0.069 (0.026, 56)
RF	0.11 (0.013, NA)	0.033 (0.013, NA)	0.048 (0.018, NA)
SQDA	0.206 (0.134, 1300)	0.021 (0.015, 2200)	0.04 (0.041, 500)
DLDA2	0.128 (0.026, 3400)	0.033 (0.01, 6600)	0.068 (0.02, 7800)
DQDA2	0.205 (0.066, 3100)	0.049 (0.022, 5900)	0.089 (0.027, 6100)
**Methods**	**BC**	**KC**	**CC**
DLDA	0.035 (0.017, 50)	0.028 (0.018, 50)	0.006 (0.008, 50)
DQDA	0.018 (0.009, 50)	0.037 (0.03, 50)	0.004 (0.006, 50)
NN	0.021 (0.013, 50)	0.031 (0.019, 50)	0.005 (0.009, 50)
SVM	0.018 (0.012, 50)	0.028 (0.018, 50)	0.004 (0.006, 50)
SCRDA	0.045 (0.019, 452)	0.047 (0.011, 78)	0.023 (0.014, 49)
RF	0.027 (0.013, NA)	0.025 (0.014, NA)	0.011 (0.011, NA)
SQDA	0.021 (0.008, 2800)	0.009 (0.005, 6100)	0.007 (0.008, 5900)
DLDA2	0.036 (0.015, 8000)	0.039 (0.007, 10200)	0.02 (0.014, 11700)
DQDA2	0.069 (0.033, 7400)	0.045 (0.035, 9600)	0.021 (0.014, 10200)

The reported numbers in each table entry in the form of *a (b,c)* mean: *a* is the average prediction error, *b* is the standard deviation, and *c* is the median number of predictors selected.

## Discussion

In this paper, we have proposed a sparse version of QDA to take into account differing genetic networks across different classes in sample classifications. When the proposed method, SQDA, was compared with six commonly used classification methods on both simulated data and real data, we found that SQDA has good performance, especially when different classes have different covariance matrices.

In order to alleviate the intensive computation burden, we have imposed the block-diagonal structure assumption on the covariance matrix. We further assumed that the block size is the same for all blocks. Although this assumption seems too simple to characterize the complex relationships among genes, it does offer a good compromise between the diagonal covariance matrix assumption made in previous methods and the more complex covariance matrix structures that may require much more data to model. The better performance of this approach has been demonstrated in both simulations and, more importantly, the TCGA datasets.

In our comparison of SQDA with six other classification methods on simulated and real data, we consider block size of 100 and error margin of 0.05 based on the simulation. In practice, the block size and error margin can be selected using cross-validation. However, due to the small sample size in real data, tuning too many parameters using cross-validation may lead to more variations in the results. As sample size increases, cross validation-based or other methods may be used to select block size and error margin to improve the performance of our method.

Instead of determining the blocks based on two sample *t* statistics and using the same size for each block, a data-driven way of determining the blocks might be better. For example, as suggested in [23], hierarchical clustering based on the correlation matrix summarized across all classes could be used to determine the blocks, where the number of clusters (i.e. blocks) is determined using cross-validation. However, when using cross-validation to choose the number of clusters, the cluster size (i.e. block size) could be larger than 1000, which makes it computationally prohibitive to tune the sparsity parameters in estimating the covariance matrix for those large blocks.

We have considered binary classification for both simulations and real data analysis. We note that SQDA can be easily extended to multi-class classification problems.

## Conclusions

In summary, we have proposed a sparse version of QDA, which has better or similar performance than commonly used classification methods based on both simulated data and real data. We believe SQDA will prove useful for classification in genomics studies and other research settings, where covariances differ among classes. A R package, SQDA, can be used to perform sparse quadratic discriminant data analysis and is freely available on CRAN (http://cran.r-project.org).

## Methods

In this section, we will first review the existing methods and then introduce our method.

### LDA, QDA, DLDA, and DQDA

Assume we collect data from *n* samples with each sample having *p* features. We further assume that the samples are drawn from *K* classes. Let *Y* denote the class label, i.e. *Y*_*i*_=*k* means the *i* ^*th*^ sample belongs to the *k*_*th*_ class, where *k*=1,…,*K*. Let *X* denote the vector of features, that is *X*_*i*_ is a *p*-dimensional vector with each coordinate being the value for the corresponding feature for *i* ^*th*^ sample. In LDA and QDA, the features in each class are assumed to follow a multivariate Gaussian distribution, that is

(3) $${} X_{i} | Y_{i}=k\sim N\left(\mu_{k},\Sigma_{k}\right)  $$

(4) $${} {\fontsize{8.8pt}{9.6pt}\selectfont{\begin{aligned} f(X_{i} | Y_{i}=k) =\frac{1}{\sqrt{(2\pi)^{p} |\Sigma_{k}|}}\exp\!\left(\!-\frac{1}{2} (X_{i}-\mu_{k})'\Sigma_{k}^{-1}(X_{i}-\mu_{k})\!\right)\!. \end{aligned}}}  $$

Thus, we can assign the *i*_*th*_ sample to one of *K* based on the maximum likelihood rule, that is

(5) $$\begin{array}{@{}rcl@{}} {}Y_{i}&=&\underset{k=1,\ldots,K}{\arg\, \max}\, f(X_{i} |Y_{i}=k) \end{array} $$

(6) $$\begin{array}{@{}rcl@{}} {}&=&\underset{k=1,\ldots,K}{\arg\, \max} \log f(X_{i} |Y_{i}=k). \end{array} $$

In LDA, the covariance matrices are assumed to be the same for all classes, that is *Σ*_*k*_=*Σ*, for *k*=1,…,*K*, and hence the maximum likelihood rule can be simplified to

(7) $$\begin{array}{@{}rcl@{}} {}Y_{i}&=&\underset{k=1,\ldots,K}{\arg\, \min} \left(X_{i}-\mu_{k}\right)'\Sigma^{-1}\left(X_{i}-\mu_{k}\right) \end{array} $$

(8) $$\begin{array}{@{}rcl@{}} {}&=&\underset{k=1,\ldots,K}{\arg \, \min}\, \mu_{k}' \Sigma^{-1} \mu_{k} -2\mu_{k}'\Sigma^{-1}X_{i}. \end{array} $$

In practice, the parameters *μ*_*k*_ and *Σ* are unknown and need to be estimated. In general, they are estimated by the sample mean ($\bar {x}_{k}$) and sample covariance matrix ($\hat {\Sigma }$) respectively. In the high-dimensional setting where *p*≫*n*, the sample covariance matrix ($\hat {\Sigma }$) is singular.

In DLDA, the covariance matrix (*Σ*) is estimated by the diagonal common sample covariance matrix, that is $\hat {\Sigma }$ is diagonal with each diagonal element being the pooled sample variance of the corresponding predictor.

In DQDA, the covariance matrix for each class (*Σ*_*k*_) is estimated by the diagonal sample covariance matrix based on the samples belonging to class *k*.

### SCRDA

In SCRDA, the covariance matrix is estimated through a linear combination of sample covariance matrix and the identity matrix, that is

(9) $$ \tilde{\Sigma}=\alpha \hat{\Sigma}+ (1-\alpha)I_{p},  $$

where *α*∈[ 0,1].

Alternatively, shrinkage can be applied to the correlation matrix, that is

(10) $$ \tilde{R}=\alpha \hat{R}+ (1-\alpha)I_{p},  $$

where *α*∈[ 0,1], $\hat {R}$ is the sample correlation matrix.

In addition to the shrunken covariance matrix estimator, the means for each class can also be estimated through shrinkage based on the “nearest shrunken centroids”, that is

(11) $$ \bar{x}^{*}_{k}=\text{sgn}\left(\bar{x}^{*}_{k}\right)\left(|\bar{x}^{*}_{k}| - \Delta\right)_{+},  $$

where $\bar {x}^{*}_{k}=\tilde {\Sigma }^{-1}\bar {x}_{k}$ and $\bar {x}_{k}$ is the sample mean for class *k*.

In practice, (*α*,*Δ*) need to be tuned based on cross-validation. If there are several pairs of (*α*,*Δ*) having the same cross-validation error, we use the “MIN-MIN” rule mentioned in [8] to choose their parameters. This is accomplished using the R package *rda*.

### NN (nearest neighbor)

In NN, a new sample with predictor *X* ^′^ is classified into one of the *K* classes based on the class labels of the *h* known samples that are closest to the new sample defined in terms of euclidean distance defined over all the predictors, where *h* is a pre-defined integer. The class label *Y* ^′^ for the new sample is usually inferred by the majority vote from these *h* selected samples, that is

(12) $$ Y'=\underset{k=1,\ldots,K}{\arg\, \max} \sum_{i\in S}\delta(Y_{i}=k)  $$

where *δ*() is the indictor function and *S* is the index set.

In our comparison, *h* is usually chosen to be 3, a common practice in genomics data analysis. In our comparisons, we used the *knn* function implemented in R package *class*.

### SVM

In the binary classification problem, i.e. *K*=2, SVM aims to find a hyperplane that can separate the two classes. If perfect separating hyperplanes exist, that is the two class can be separated perfectly, then the maximum-margin hyperplane is chosen, which maximizes the total distance of the closest point in each class to the hyperplane. If no perfect separating hyperplane exists, the hyperplane maximizing the margins while keeping the misclassification rate as low as possible is chosen.

In SVM, the kernel function has to be predefined, which basically determines the shape of the hyperplanes. Popular kernel functions include linear kernel function, polynomial kernel function, and Gaussian radial basis function. When there are more than two classes, a single multi-class problem is often decomposed into multiple binary classification problems.

In our comparison, we used SVM with the linear kernel, because the sample size is small. We used the *svm* function in R package *e*1071 in our comparisons.

### RF

The basic idea of RF is to grow a forest of classification trees based on the training data and then use the classification trees to classify testing data. More specifically, for a given training dataset, *B* bootstrapped datasets are used to build R decision trees where a random subset of predictors are evaluated at each node [24]. The Random Forest, which consists of *B* prediction trees, is used for classifying future samples. For a test sample, each prediction tree will assign it to one of the *K* classes and the class label of this sample is then determined by majority vote from the *B* decision trees.

We used the R package *randomForest* in our comparisons.

### Proposed method

In this article, we propose a modified version of QDA with sparse estimation of the covariance matrix. We call it SQDA.

In SQDA, we adopted the method introduced in [25] to obtain a sparse estimator of the covariance matrix. The sparse estimate for the correlation matrix is first obtained by the following optimization criterion and then transformed back to the original scale using the sample variance, which yields a sparse estimate for the covariance matrix.

(13) $$  \tilde{R}_{\lambda} = \underset{R\succ 0}{\arg \, \min}\, {|| R-\hat{R}||}_{F}^{2}/2 -\tau \log |R|+\lambda {|R^{-}|}_{1}  $$

where ||·||_*F*_ is the Frobenius norm, |·|_1_ is the *L*_1_ norm, *τ* is a fixed small value, *λ* is a tuning parameter, and *R* ^−^ means *R* with diagonal elements set to 0.

However, it is time consuming to estimate the covariance matrix for extremely large *p* based on Equation 13. To reduce computational burden, we assume covariance matrices for all classes have block-diagonal structure to allow us to estimate the covariance matrices one block at a time. The idea of using block-diagonal structure to approximate the inverse of covariance matrix has been applied in LDA by [26]. However, the inverse of covariance matrix still has to be estimated in their method, which is time consuming.

Under the block-diagonal structure assumption for the covariance matrix, the order of features matters, that is we need to know which features form a block. In our algorithm, we sort the genes based on the absolute two sample t statistics and the blocks are chosen from the top to the bottom with each block having the same size. There were a couple of reasons that led us to choose our approach. First, genes with similar expression level differences across the classes are grouped together based on two sample t statistics so that genes with similar informativeness on classifications are likely to be selected or excluded together. Second, genes that are highly correlated are likely to have similar absolute values in terms of two sample t statistics, so if the covariance matrix needs to be approximated by a block-structured one, then grouping genes by t statistics is more likely to result in a better approximation of the true covariance matrix.

**Figure 3:**
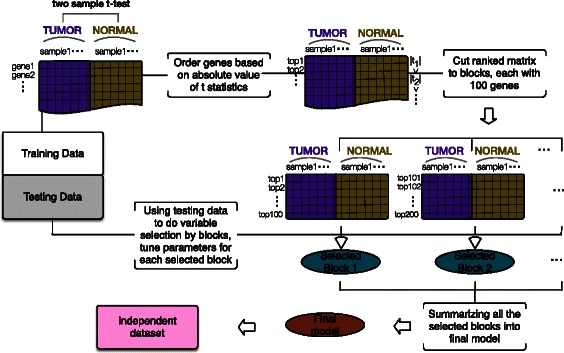
**Workflow of SQDA.** This figure decribes the general workflow of SQDA based on a toy example of classifications of tumor and normal samples.

To illustrate the workflow of our proposed method (as shown in Figure 3), let us consider the example in the classification of tumor and normal tissues based on gene expression profiles. First, the two sample *t* statistics based on the training data are calculated for each gene. The genes are then ordered based on the absolute values of the t statistics. To be computationally more efficient, an optional step here is to do variable selection by blocks (see section Variable selection). Secondly, the covariance matrices are estimated one block at a time with block size 100 from the top to the bottom. Lastly, the final prediction model is the quadratic discriminant analysis model with the covariance matrices obtained from all the blocks.

For each block in SQDA, we use the same *λ* for all the classes and choose the value of *λ* through cross-validation. When several *λ* values lead to the same cross validation error, we choose the minimum value for *λ*. To perform sparse estimation of the covariance matrix, the R package *PDSCE* is used.

### Variable selection

We use multiple gene expression data to compare the performance of different classification methods. For gene expression datasets, the number of genes is usually on the scale of thousands while the number of samples is on the scale of tens. Thus, it may be better to perform gene pre-screening to improve classification performance. In this paper, we use the R package *limma* to rank the genes based on the empirical Bayes-based *t* statistics (for binary classification problem) or the *F* statistics (for multi-class classification problem).

For DLDA, DQDA, NN, and SVM, we use five-fold cross validation based on the training sample to pick the number of top genes used for prediction ranging from 50 to 2000 with step size 50.

For SCRDA and RF, since they can perform variable selection and classification simultaneously, no variable selection step is performed.

For our method, SQDA, we do variable selection by blocks. We set the sparsity parameter *λ* to be 0.2 and calculate cross-validation error for each block and then we choose the blocks with cross validation errors less than error margin + the smallest cross-validation error, where error margin can be any number from 0 to 0.5 and usually 0.05, 0.10 and 0.15 are used.

For DLDA and DQDA, we also perform variable selection by blocks, leading to DLDA2 and DQDA2, that is to use the same procedure as our method to do variable selection except that the sparse estimation of covariance matrix is replaced by diagonalized estimators for covariance matrix.
